# Study of deactivation in mesocellular foam carbon (MCF-C) catalyst used in gas-phase dehydrogenation of ethanol

**DOI:** 10.1038/s41598-021-91190-7

**Published:** 2021-06-03

**Authors:** Yoottapong Klinthongchai, Seeroong Prichanont, Piyasan Praserthdam, Bunjerd Jongsomjit

**Affiliations:** Center of Excellence On Catalysis and Catalytic Reaction Engineering, Department of Chemical Engineering, Faculty of Engineering, Chulalongkorn University, Bangkok, 10330 Thailand

**Keywords:** Environmental sciences, Chemistry, Energy science and technology, Engineering, Materials science

## Abstract

Mesocellular foam carbon (MCF-C) is one the captivating materials for using in gas phase dehydrogenation of ethanol. Extraordinary, enlarge pore size, high surface area, high acidity, and spherical shape with interconnected pore for high diffusion. In contrary, the occurrence of the coke is a majority causes for inhibiting the active sites on catalyst surface. Thus, this study aims to investigate the occurrence of the coke to optimize the higher catalytic activity, and also to avoid the coke formation. The MCF-C was synthesized and investigated using various techniques. MCF-C was spent in gas-phase dehydrogenation of ethanol under mild conditions. The deactivation of catalyst was investigated toward different conditions. Effects of reaction condition including different reaction temperatures of 300, 350, and 400 °C on the deactivation behaviors were determined. The results indicated that the operating temperature at 400 °C significantly retained the lowest change of ethanol conversion, which favored in the higher temperature. After running reaction, the physical properties as pore size, surface area, and pore volume of spent catalysts were decreased owing to the coke formation, which possibly blocked the pore that directly affected to the difficult diffusion of reactant and caused to be lower in catalytic activity. Furthermore, a slight decrease in either acidity or basicity was observed owing to consumption of reactant at surface of catalyst or chemical change on surface caused by coke formation. Therefore, it can remarkably choose the suitable operating temperature to avoid deactivation of catalyst, and then optimize the ethanol conversion or yield of acetaldehyde.

## Introduction

The renewable energy has high impact to the world in the last decade, especially in many countries, in order to use the renewable clean fuel with eco-friendly environment such as bioethanol. As known, one of the crucial bioethanol production processes is the fermentation of sugars as the major sources from sugar cane and starch, which is uncomplicated process in the production^1–5^. At present, ethanol is not only interesting in fields of alternative fuel or even blending of alcohols with gasoline or biodiesel fuels^6^, but also as attentive feedstock to produce essential origination chemicals for chemical industries such as acetic acid, ethyl acetate, butanol, acetaldehyde, etc.^7^. In this research, we emphasized our consideration on the feasibility in direct production of acetaldehyde from ethanol via catalytic dehydrogenation, which is considered as cleaner foresight technology. Some researchers previously investigated the reaction of ethanol dehydrogenation to acetaldehyde^8,9^, and this reaction undergoes using proper catalysts as follows (Eq. 1):

1 $$CH_{3}CH_{2}OH\leftrightarrow CH_{3}CHO+ H_{2}$$

In fact, there are different types of carbon catalysts used in dehydrogenation process. Previously, Liu et al*.*^10^ reported that ordered mesoporous carbon catalyst essentially catalyzed the dehydrogenation of propane to propylene with high activity. Later, Ob-eye et al*.*^11^ also reported that ethanol dehydrogenation to acetaldehyde apparently occurred using activated carbon-promoted with cobalt (Co) having very high selectivity to acetaldehyde. Furthermore, activated carbon from bacterial cellulose could be employed as the catalyst in ethanol dehydrogenation with the high catalytic activity^12^. Thus, ethanol conversion over carbon materials is captivating. It is also recognized that the mesocellular foam carbon (MCF-C) is one of the robust carbon catalysts, which can be employed in ethanol dehydrogenation in order to produce acetaldehyde. This is owing to its appropriate physicochemical properties such as desired pore characteristics and acid–base properties as reported in our previous study^13^. In addition, the structure of MCF-C is well defined as the interconnected pore and large pore size, which not only provided higher diffusion, but also accorded high activity as conversion or even selectivity. Besides, the deactivation of this catalysts is very important issue since it is related with stability of catalyst. Thus, it seems reasonable to investigate the deactivation behavior of MCF-C catalyst via ethanol dehydrogenation in order to better understand the nature of this catalyst^13^. Nevertheless, the general cause to deactivate most catalysts on ethanol dehydrogenation is derived from coke formation. For this aim, it must be discussed that there is the correlation between the decrease in the catalyst activity and the catalyst deactivation from the occurrence of the coke inside of the catalyst. In addition, this verity is a consequence of the heterogeneous nature of the coke in the catalyst, which is possibly composed of amorphous and filamentous fractions, with the cokes of amorphous structure that have a significant impact on catalyst deactivation owing to the encapsulation in the catalyst^14–16^. Thus, several procedures expected at selecting and adapting catalysts have been considered in the literature to minimize the coke deposition in the catalyst. According to Montero et al*.*^17^, they investigated the deactivation of Ni/La_2_O_3_-α-Al_2_O_3_ catalyst in ethanol steam reforming (ESR) with different operating condition as either temperature between 500 and 650 °C or space time up to 0.35 g_catalyst_h/g_EtOH_. They reported that catalyst deactivation was merely motived by coke deposition, remarkably via encapsulating coke inside of the catalyst. In addition, Morales et al*.*^18^ also investigated the difference in deactivation of Au catalyst during transformation when supported on ZnO and TiO_2_. The evidence suggested that the catalyst on ZnO demonstrated higher resistance to deactivation caused by coke formation. Therefore, the selection of catalysts in each specific reaction is captivating to exhibit either high activity or resistance to deactivation caused by coke formation. It is known that the decline in deactivation of catalyst is regularly followed by an increase in the carbon content on the catalyst surface with different conditions. To the best of our knowledge, no work in the literature has been yet reported on the deactivation behaviors MCF-C catalyst used in gas-phase ethanol dehydrogenation to acetaldehyde.

Accordingly, this research is emphasized on the effects of operating conditions such as reaction temperature and weight hourly space velocity (WHSV) on the formation of coke under mild condition. According to the study, gas-phase ethanol dehydrogenation over MCF-C catalyst was carried out in a micro fixed-bed reactor, which is possibly reasonable for the scaling-up, capacitates thermal uniformity of the catalytic bed, and moderates the deactivation via coke deposition as well. The spent catalysts under specified condition were collected after each run and characterized by nitrogen-physisorption, X-ray diffraction (XRD), scanning electronic microscopy (SEM), thermogravimetric analysis (TGA), ammonia temperature-programmed desorption (NH_3_-TPD), carbon dioxide temperature-programmed desorption (CO_2_-TPD) and Fourier transform infrared spectroscopy (FT-IR) in order to observe the changes of catalysts after being used.

## Experimental

### Materials and method

#### Materials (Chemicals)

Pluronic P123 (Sigma-Aldrich, Molar mass ∼ 5800) was used as the surfactant or template, and hydrochloric acid (HCl (98 wt%), Sigma-Aldrich) was used to catalyze in the synthesis of MCF-Si for forming the structure of materials. Furthermore, 1,3,5-trimethylbenzene (TMB, Sigma-Aldrich) was used as the swelling agent, which can expand the pore of material. The silica source was from tetraethyl orthosilicate (TEOS; 98% purity, Sigma- Aldrich). Sulfuric acid (H_2_SO_4_ (98 wt%, Sigma Aldrich) was employed as the provider of the formation of carbon layer. The etching of silica was used as sodium hydroxide (NaOH, SigmaAldrich).

#### Catalyst preparation

Mesocellular foam carbon (MCF-C) was synthesized using mesocellular foam silica (MCF-Si) as based material^13^. First, 2 g of Pluronic P123 as triblock copolymer was dissolved in 10 ml of hydrochloric acid with 65 ml of deionized water by stirring until it became homogeneous solution (ca. 1 h) at ambient temperature. After that, 5 g of 1,3,5-trimethyl benzene (TMB) as the pore expander was added into the prior solution at 40 °C, and continuously stirred for 2 h to obtain milky solution. After approaching 2 h, tetraethyl orthosilicate (TEOS) used as the silica source was consecutively added into the previous solution, and then kept rapidly stirring at the same temperature for 5 min. Consequently, the milky solution was transferred into Teflon bottle, which was followed by aging at 40 °C for 20 h. After reaching 20 h, the temperature was increased to 100 °C with the ramping rate of 10 °C/min. The white solution was filtered with 50 ml of ethanol and 100 ml of deionized water, and then dried overnight at room temperature. The white precipitate of MCF-Si was ready to be used as the based material for MCF-C synthesis. To obtain MCF-C, 1 g of MCF-Si was mixed with 0.16 ml of sulfuric acid, and also stirred it for 1 h. After that, it was dried in the oven at 100 °C for 12 h. Then, the temperature was increased to 160 °C for 12 h. The black powder was calcined at 850 °C under nitrogen flowing for 2 h with ramping rate of 1 °C/min. Next, the etching process was applied using 2 M of NaOH to eliminate the silica from the material at ambient temperature with stirring for 1 h. In addition, it was followed by washing with deionized water until the pH of filtrate was exactly unchanged, and dried overnight at room temperature. Finally, the MCF-C was ready to use.

#### Characterization of catalyst

##### Nitrogen-physisorption

Nitrogen-physisorption was used to measure the pore size, surface area, and pore volume of samples using Micromeritics ChemiSorb 2750 Pulse instrument. Measuring of Brunauer-Emmet-Teller (BET) isotherm equation was performed at − 196 °C, and the samples were degassed with heating in the vacuum at ambient temperature to 120 °C for 16 h. In addition, Barrett-Joyner-Halenda (BJH) method based on the Kelvin equation was also employed to evaluate the pore structure of samples^19^.

##### Scanning electron microscopy (SEM)

The morphology of specimens was identified using the Hitachi S-3400N model. Link Isis Series 300 program EDX was applied to analyze the elemental distribution and composition over different catalysts.

##### X-ray diffraction (XRD)

XRD was used to estimate the crystalline framework of samples using a Siemens D 5000 X-ray diffractometer having CuK_α_ radiation with Ni filter in the range of 2θ between 1 and 60 with 0.04 resolution. The scan rate was applied at 0.5 s/step.

##### Thermogravimetric analysis (TGA)

TGA was operated using TA instrument SDT Q600 analyzer (USA). The sample of 4–10 mg was used in the temperature operation range between 0 and 1000 °C with heating rate of 2 °C/min using air as carrier gas.

##### Fourier transform infrared (FT-IR) spectroscopy

The functional groups of specimens were analyzed using the FTIR spectroscopy. The signal absorption spectra were obtained using Nicolet 6700 FTIR spectrometer in the wavenumber range of 400–4000 cm^-1^.

##### Ammonia temperature-programmed desorption (NH_3_-TPD)

The acidity and acid strength of catalysts were examined applying Micromeritics Chemisorb 2750 Pulse Chemisorption System^13^. First, 0.1 g of catalyst was preheated with helium at 200 °C. Then, ammonia was adsorbed at 40 °C for 1 h. After that, the physisorbed ammonia was desorbed under helium gas flow until the baseline level was reached to be constant. The chemisorbed ammonia was removed from active sites by raising the temperature from 30 to 500 °C under a helium flowing at 40 ml/min, with a heating rate of 10 °C/min. The thermal conductivity detector (TCD) as a function of temperature was adapted to estimate the amount of ammonia in effluent.

##### Carbon dioxide temperature-programmed desorption (CO_2_-TPD)

CO_2_-TPD technique was used to analyze the basicity and its strength of samples catalyst by using Micromeritics Chemisorb 2750 automated system. The sample of 0.1 g was packed among the center of the quartz cell and preheated with temperature of 450 °C under helium with flow rate of 25 ml/min for 1 h to eliminate moisture and impurity. After that, the sample was directly saturated with _CO2_ and evacuated by helium with flow rate of 35 ml/min for 30 min at 40 °C. Then, TPD was operated from 40 to 500 °C with heating rate of 10 °C/min. Finally, thermal conductivity detector (TCD) was used to determine the amount of CO2 in effluent gas as a function of desorbed temperature.

#### Catalytic test

The deactivation behavior of catalyst was determined using the ethanol dehydrogenation test apparatus using a fixed-bed continuous flow glass tube microreactor. Starting with 1 g of catalyst sample (MCF-C) and 0.05 g of quartz wool bed were packed inside of the central glass tube reactor, which was placed inside of the electric furnace. The pretreatment at 200 °C under nitrogen flowing for 1 h was conducted to remove the humidity on the surface of target catalyst. Then, the liquid ethanol was vaporized at 120 °C with nitrogen gas at 60 ml/min using controlled injection with a single syringe pump with the volumetric flow rate of ethanol feeding at 0.397 ml/h. To obtain the spent samples (deactivation catalysts), the gas stream was fed into the reactor with weight hourly space velocity (WHSV) in the desired feeding of 3.11 g_ethanol_ /g_cat_.h. Furthermore, the considerable operating temperature range was at 300, 350, and 400 °C under atmospheric pressure. The gaseous products were analyzed by a Shimadzu (GC-14B) gas chromatograph with flame ionization detector (FID) using capillary column (DB-5) at 150 °C. While the reaction test^13^ was operated, the results were repeatedly recorded at least 3 times for each temperature. After running different operating temperatures of 300, 350, and 400 °C, the spent catalysts were denoted to MCF-C SP300, MCF-C SP350, and MCF-C SP400, respectively.

The values of ethanol conversion, selectivity of acetaldehyde, and yield of acetaldehyde were diagnose using these following Eqs. (2), (3), and (4), respectively.

2 $$ {\text{Ethanol}}\,{\text{conversion:}}\quad X_{EtOH} (\% ) = \frac{{n_{EtOH} (in) - n_{EtOH} (out)}}{{n_{EtOH} (in)}} \times 100 $$

3 $$ {\text{Selectivity}}\,{\text{of}}\,{\text{acetaldehyde:}}\quad S_{i} (\% ) = \frac{{n_{i} }}{{\sum n_{i} }} \times 100 $$

4 $$ {\text{Yield}}\,{\text{of}}\,{\text{acetaldehyde:}}\quad Y_{i} (\% ) = \frac{{X_{EtOH} \times S_{i} }}{100} $$

## Results and discussion

**Figure 1:**
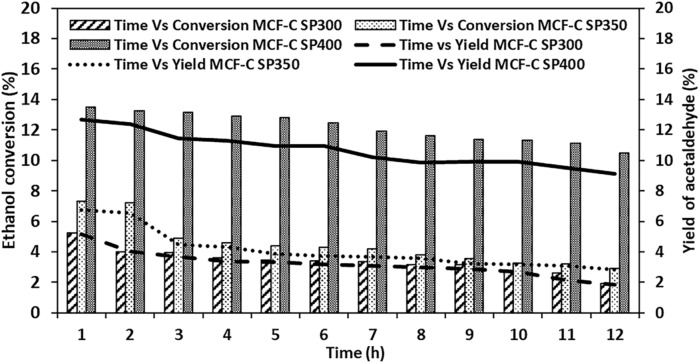
Ethanol Conversion and yield of acetaldehyde of all catalysts with regarding to ethanol dehydrogenation.

Catalytic behavior was tested via the influence of temperature during time on stream on catalytic behavior of ethanol dehydrogenation. The Effect of different temperature between 300 and 400 °C during time on stream of 12 h on the deactivation behavior of catalysts was investigated and the results are illustrated in Fig. 1. In first hour of the reaction test, the operating temperature at 400 °C exhibited the highest ethanol conversion of ca. 13.5% followed by the ones at 350 °C (ca. 7.32%), and 300 °C (ca. 5.24%). The evidence suggested within first hour of operating system that the increased ethanol conversion from 300 to 400 °C was dependent on increased temperature since dehydrogenation is endothermic reaction^20–22^, which was directly responded to the Eq. (1). In addition, yield of acetaldehyde also simultaneously increased from 5.15 to 12.68% with increasing of ethanol conversion from 300 to 400 °C. However, there was an effect of the high temperature with the lower selectivity of ethanol to acetaldehyde. During 12 h, the ethanol conversion with operating temperature of 400 °C slowly declined with time on stream from the first hour until 12 h. In addition, yield of acetaldehyde at this temperature was insignificantly changed during 12 h with percentage of ethanol change of 22.3%. This suggested that the ethanol dehydrogenation at 400 °C for 12 h was essentially stable up on the obtained values of ethanol conversion or yield of acetaldehyde.

However, with lower operating at temperatures of 350 °C and 300 °C for 12 h, ethanol conversion decreased with values of ca. 7.33–2.93% and 5.24–1.92%, respectively. From these results, it revealed that MCF-C catalyst tended to rapidly deactivate at lower temperature i.e. 300–350 °C, especially the period operating time between 1 and 3 h. In addition, this evidence suggested that the decreasing of catalytic activity with long period operation was likely occurred by deactivation of catalyst owing to the possibility of the coke formation, which affected to the active sites as acid or basic sites on the surface of catalysts, particularly the reaction temperature at 300 °C with the highest changing of percentage of ethanol conversion with value ca. 63.2%. This phenomenon suggested that the ethanol dehydrogenation reaction might not favor the low temperature due to the high coke formation, which might block the pathway inside of the catalyst. In addition, the active sites on catalyst surface also become inactive sites owing to the cover of the coke on its sites, which inhibit the catalytic activity from ethanol dehydrogenation. In addition, the change of the selectivity of acetaldehyde is illustrated in Table S1. The result showed that at operating temperature of 300 °C, it could significantly maintain the change of the selectivity of acetaldehyde within the lowest value of 1.57%. This suggested that existence of the coke formation might be from other by-products such as ethylene or ethyl acetate^23^, which did not insignificantly affect the active site for ethanol dehydrogenation to acetaldehyde. On the contrary, the selectivity of acetaldehyde decreased with increasing of the operating temperature, indicating that the higher amount of the by-products was found or decomposition of acetaldehyde occurred. However, there was an insignificant change in the selectivity of acetaldehyde in each temperature. Thus, it is quite surprising and more characterization techniques are crucial for investigation the coke formation on the spent catalyst, which notably causes to the lower catalytic activity.

**Figure 2:**
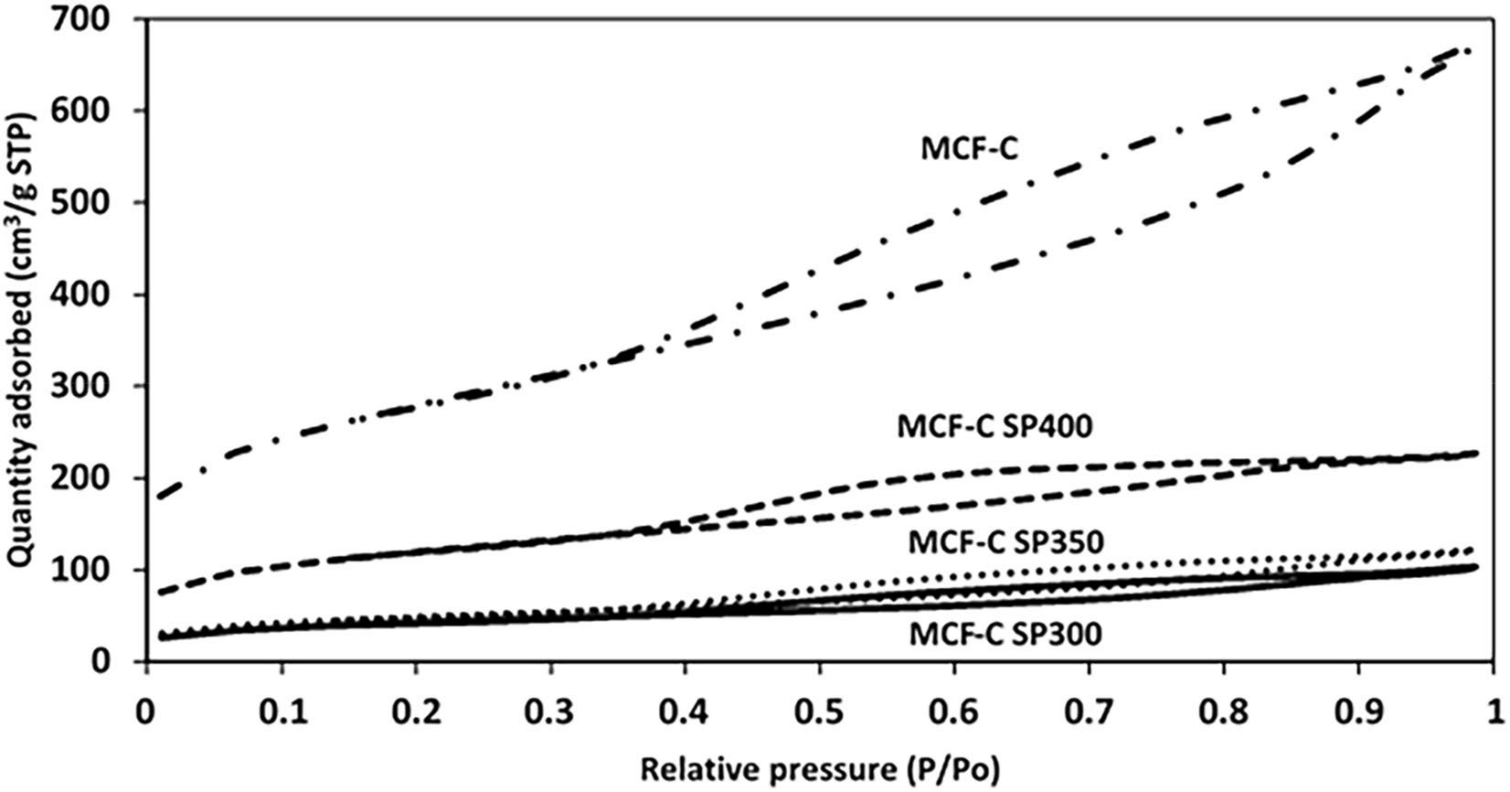
Nitrogen adsorption/desorption isotherms of all stability testing conditions (MCF-C, MCF-C SP300, MCF-C SP350, and MCF-C SP400).

The Characterization on the textural properties of catalysts was investigated in the differences on the textural properties between the fresh and spent MCF-C catalyst were elucidated using N_2_ physisorption and SEM/EDX measurement. In fact, all characterization techniques were conducted for the fresh MCF-C catalyst and spent catalysts after being used for 12 h in the reaction tests under three operating temperatures including 300, 350 and 400 °C. Thus, there were four catalyst samples in each technique to consider. First, the adsorption/desorption isotherms obtained from the N_2_ physisorption of fresh and other three spent catalysts are illustrated in Fig. 2. As seen, all catalysts exhibited the type IV (IUPAC classification) of mesoporous structure with hysteresis loop^24^. This indicated that under these specified dehydrogenation conditions; all spent MCF-C catalysts apparently retained the traditional textural structure from MCF-C as fresh catalyst^24^. In addition, MCF-C SP300, MCF-C SP350, and MCF-C SP400 demonstrated the hysteresis loop of H4 type with the narrow slit-like pore shape^24^.

**Scheme 1:**
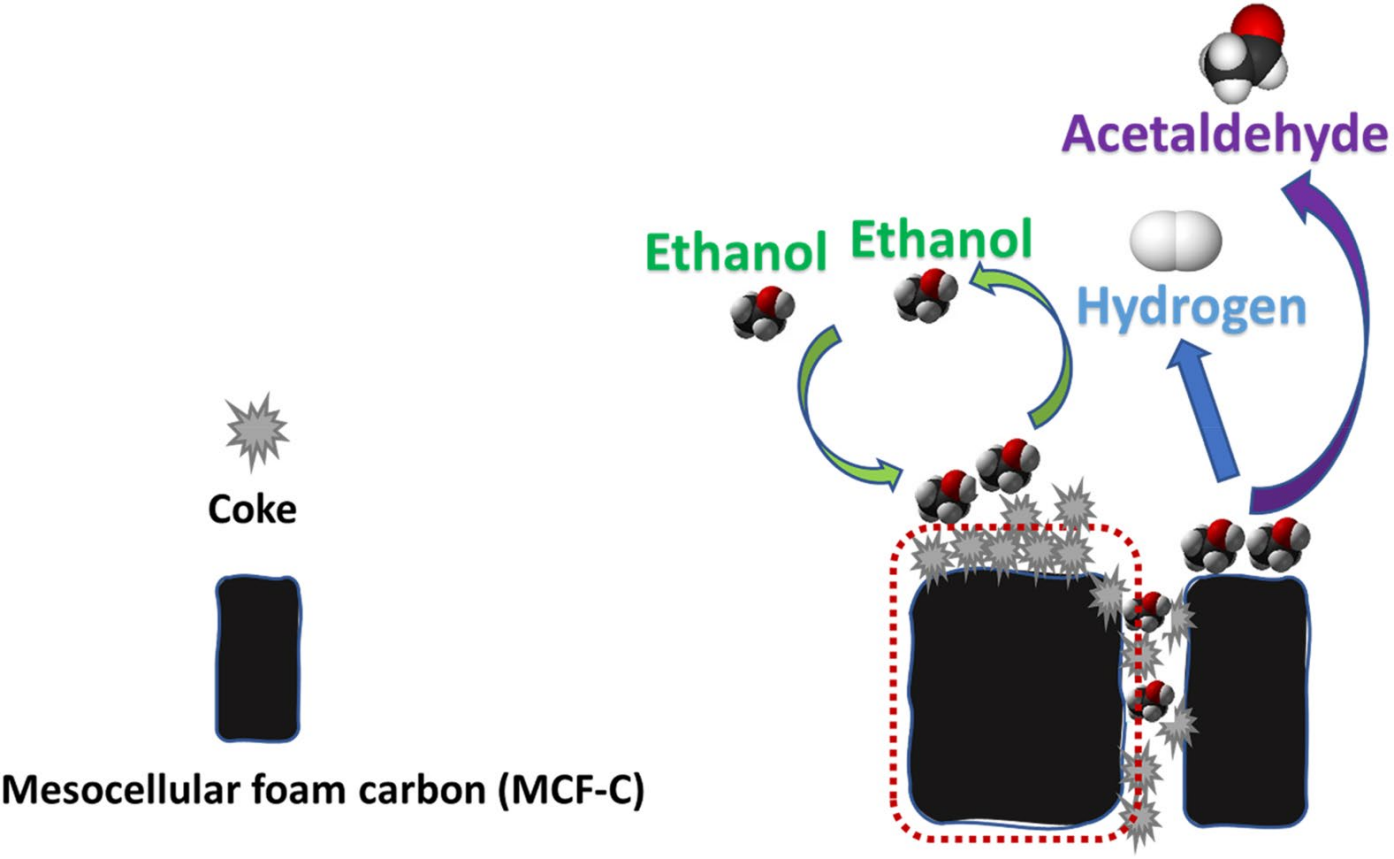
Coke formation on the surface catalysts.

This result probably suggested that coke formation likely occurred and deposited inside the pore, especially at low operating temperature such as MCF-C SP300 due to its conformation of almost nonexistence of the hysteresis loop. On the other words, MCF-C SP300 exhibited the highest coke formation, which was not reasonable to operate the ethanol dehydrogenation at this temperature. Furthermore, the pore size, surface area, and pore volume of all spent catalysts also decreased from the fresh catalyst after the test for 12 h as seen in Table 1, which confirmed that coke was probably deposited in the pore of the catalyst, especially temperature of 300 °C with the highest decreasing of physical properties. In addition, this evidence indicated that the coke formation at low temperature of 300 °C likely blocked the pathway of diffusion of reactant to the surface catalyst, which directly affected to catalytic ability of MCF-C on ethanol dehydrogenation as seen in Scheme 1. Thus, one of the crucial factors causing lower catalytic activity was the coke formation owing to the effect from pore blocking.

**Table 1:** Physical properties of the fresh and spent catalyst with different reaction temperatures.

Materials	Surface area (m^2^/g)	Average pore size (nm)	Average pore volume (cm^3^/g)
MCF-C	920.51	4.96	1.03
MCF-C SP300	312.86	2.96	0.17
MCF-C SP350	439.36	4.19	0.36
MCF-C SP400	602.16	4.27	0.48

**Figure 3:**
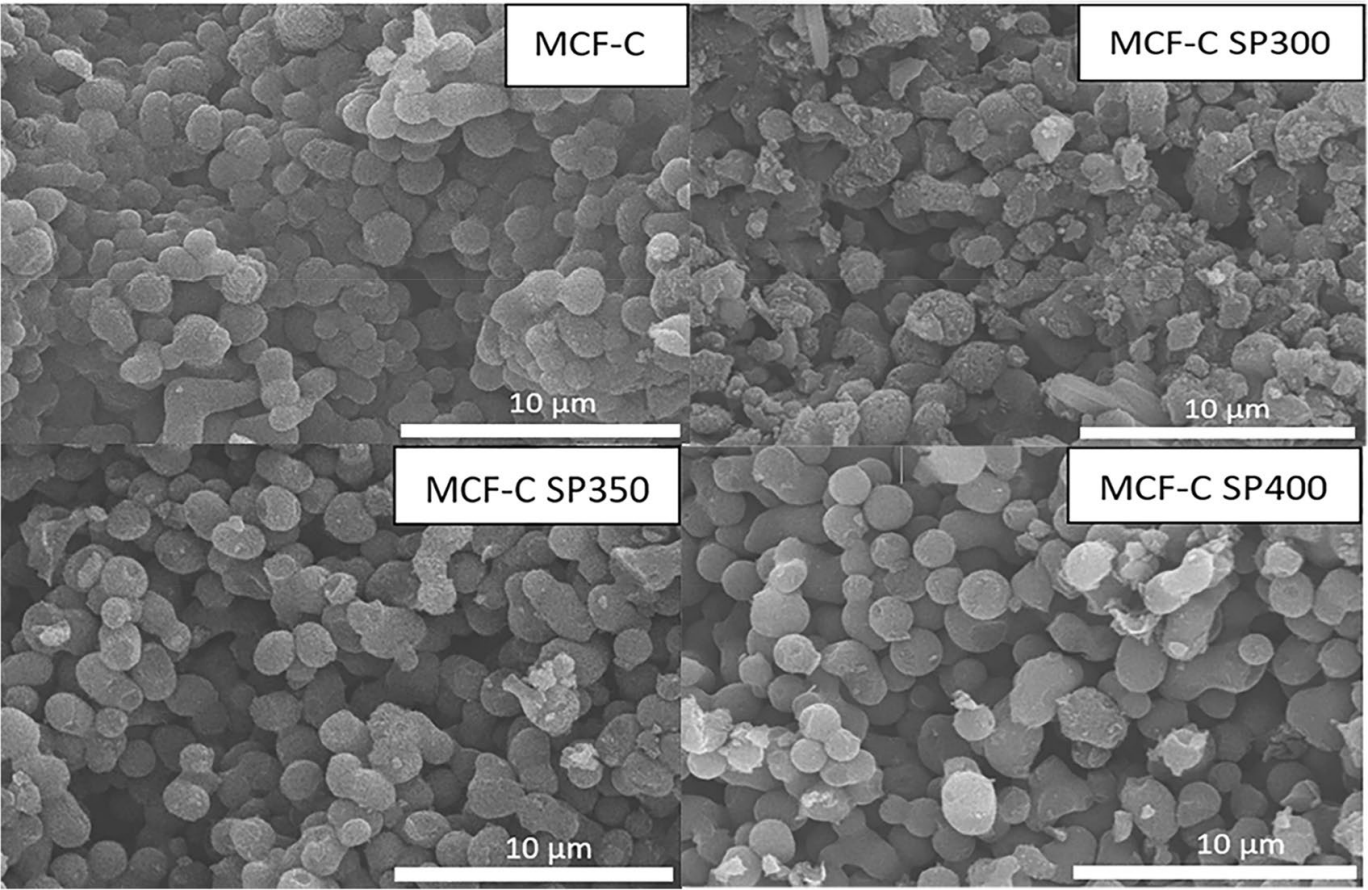
Low magnification SEM image of MCF-C, MCF-C SP300, MCF-C SP350, MCF-C SP400.

In order to verify the coke formation, SEM is one of the most powerful techniques to explore the textural property of fresh and spent catalysts as seen in Fig. 3. From SEM images of spent catalysts, it revealed that the particle size of these catalysts did not significantly change from fresh catalyst having value of ca. 1.26–1.32 µm (n = 100). This indicated that the different operating temperatures and coke formation had no effect on the particle size of these spent catalysts. However, it can be observed the emergence of the coke particles, which possibly adhered and encapsulated in all spent catalysts. Moreover, the apparent quantity of coke dispersed on MCF-C SP300 was high compared with other operating temperatures (MCF-C SP350 and MCF-C SP400, respectively). It was found that the high aggregation of the coke contents at the external surface of spent catalysts was observed at low temperature of 300 °C, which was corresponding to the low catalytic activity of ethanol dehydrogenation at this low temperature. Accordingly, the reasonable operating temperature on ethanol dehydrogenation to acetaldehyde would be 400 °C due to the lowest deactivation of the catalyst.

Besides, SEM–EDX from Table 2 demonstrated the atomic percent compositions of fresh and spent catalysts. The data displayed that there was the decreasing of the carbon contents from the fresh MCF-C catalyst from 93.26 wt% to the spent catalyst at 300 °C with the lowest carbon contents of 65.61 wt%. The decrease of carbon content on surface was due to the replacement with oxygen element, which occurred during ethanol dehydrogenation reaction. On the other hand, oxygen contents in spent catalysts increased in the order of MCF-C SP400 < MCF-C SP350 < MCF-C SP300. This phenomenon was probably due to the formation of products or by-products, which cannot migrate from the inside of the catalyst because of the pore blockage from the carbon encapsulation leading to inhibition of internal mass diffusion^12^. In addition, the low oxygen content of MCF-C SP400 was probably due to two main reasons: (a) the low coking accumulation amounts; (b) some oxygen complexes could desorb from the surface at such a high temperature.

**Table 2:** Amount of each element adjacent the surface of catalyst granule obtained from EDX.

Materials	Amount of weight on surface (wt%)		
	C	O	Si
MCF-C	93.26	6.33	0.41
MCF-C SP300	65.61	33.56	0.83
MCF-C SP350	76.45	22.63	0.92
MCF-C SP400	87.39	11.75	0.86

**Figure 4:**
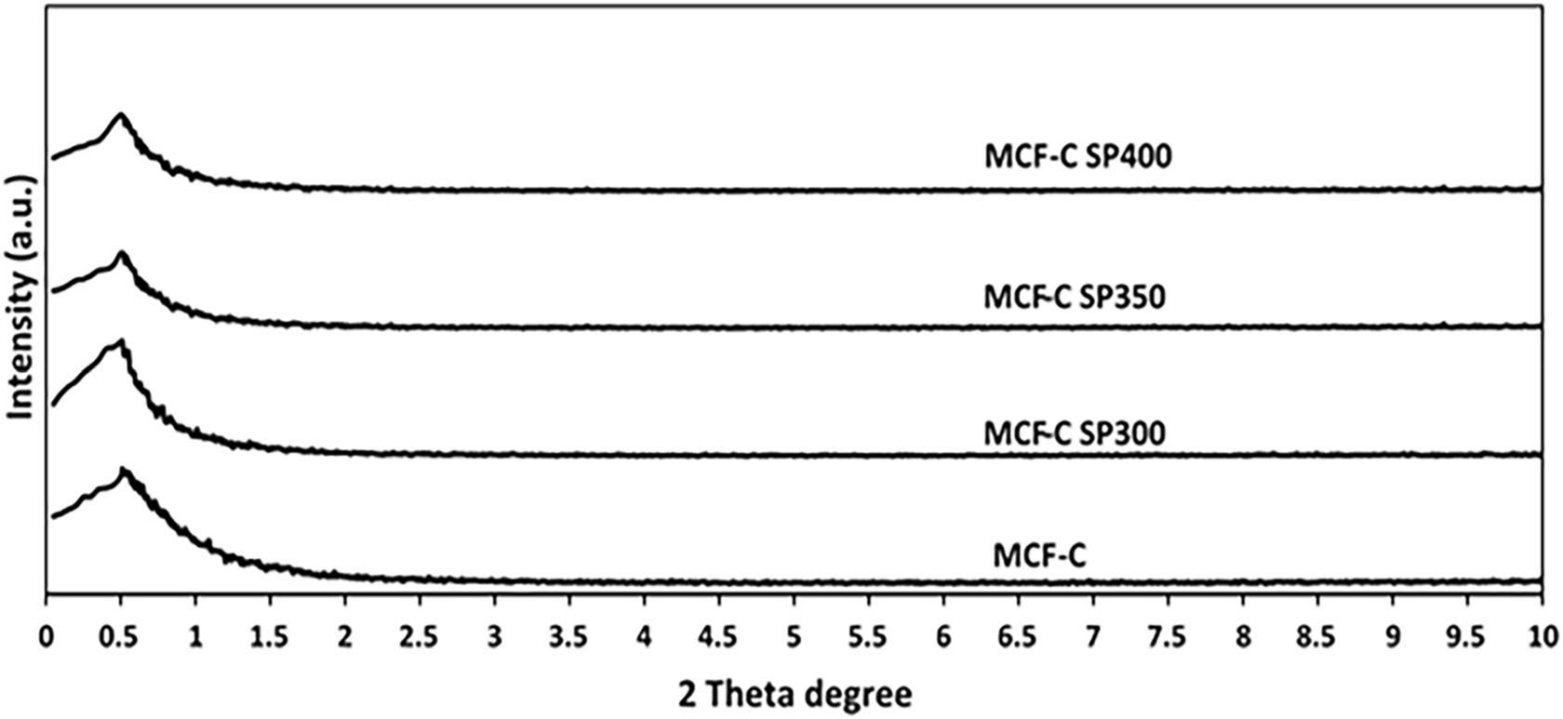
XRD pattern of the fresh and spent catalysts.

X-ray diffraction (XRD) patterns of all samples are shown in Fig. 4 in order to examine the difference of phase change of the crystal structures. All catalyst samples exhibited identical XRD peak located at 1.06° as the major crystalline phase using low-angle XRD, which is similar to previous study^13^. Nevertheless, the peak of MCF-C SP300 at the top was slightly broader than other catalysts indicating that the coke formation might softly affected the crystal structure.

**Figure 5:**
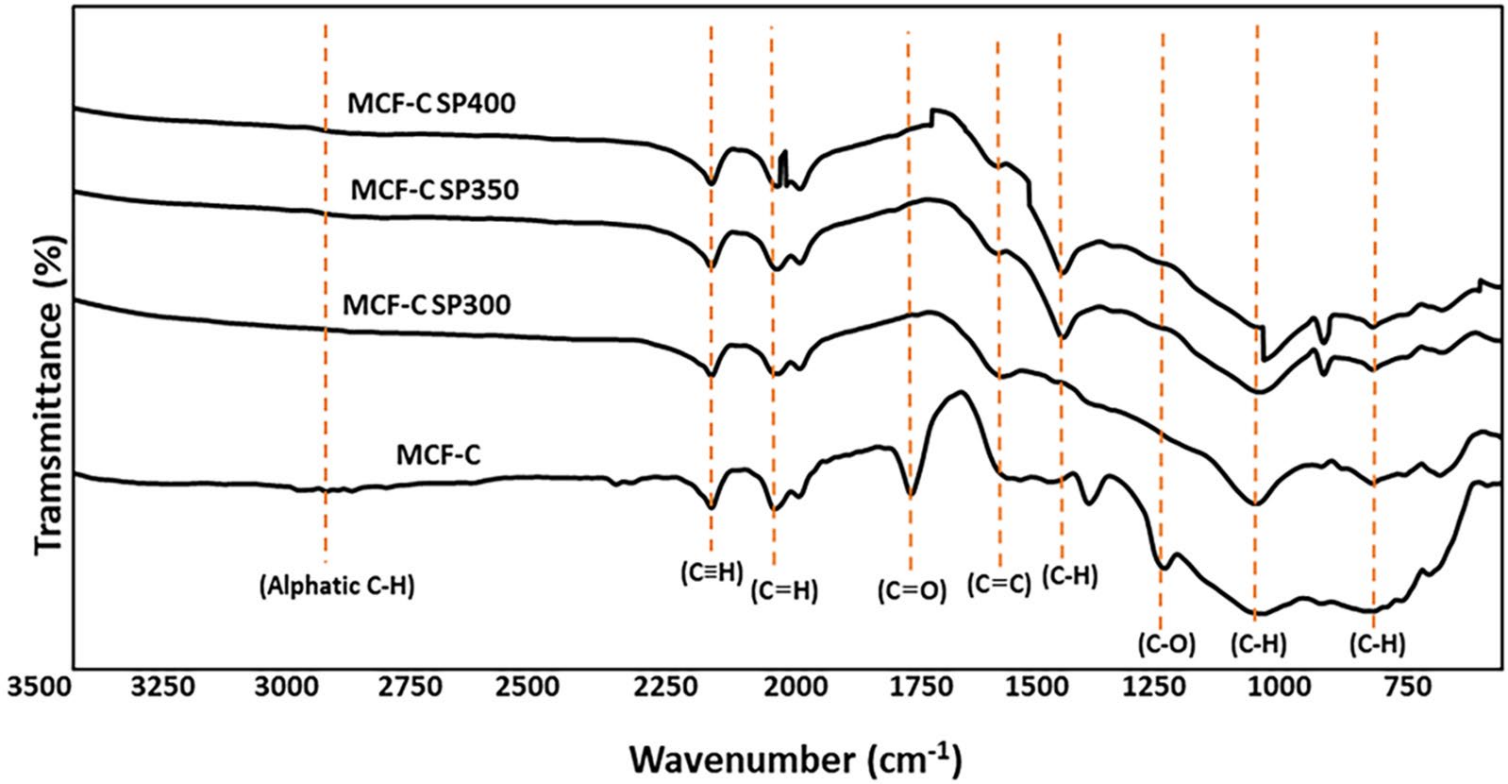
FT-IR spectra of the fresh and spent catalysts.

To identify the changes in chemical functional groups, FT-IR technique was employed on MCF-C, MCF-C SP300, MCF-C SP350, and MCF-C SP 400 as seen in Fig. 5. The IR spectrum of fresh MCF-C catalyst was well accorded with that reported in the literature^13^, having eight IR active elementary bands encountered at 759 cm^-1^ (C–H vibrations), 1020 cm^-1^ (C–H vibrations), 1239 cm^-1^ (O–H blending), 1550 cm^-1^ (C=C stretching vibrations), 1755 cm^-1^ (C=O stretching vibrations), 2040 cm^-1^ (C=C stretching vibrations), 2150 cm^-1^ (C≡C stretching vibrations), and 2970 cm^-1^ (aliphatic C–H)^11,25–27^. For all spent catalysts, the region at 750–800 cm^-1^ was observed an increase of the peak of (C–H vibrations) suggesting coke formation, especially in MCF-C SP300 catalyst. In addition, it was also found the peak at the regions of 1000–1100 cm^-1^ (C–H vibrations) indicating the C–H band that increased with decreasing the operating temperatures. This also suggested that the presence of the coke was likely initiated when the operation temperature was low i.e. 300 °C.

**Figure 6:**
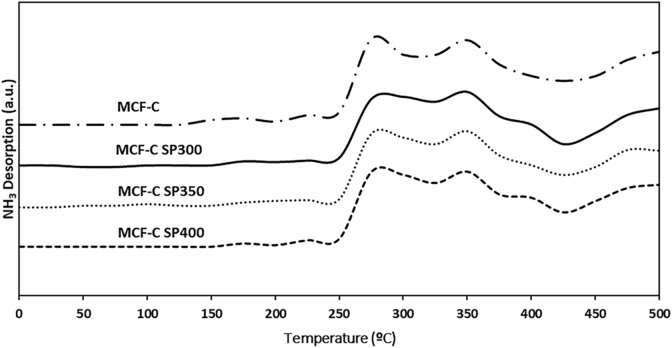
TPD-NH_3_ profile of the fresh and spent catalysts.

One of the major factors that affects the catalytic properties is acidity and acid strength^13,28,29^. Thus, ammonia temperature-programmed desorption (NH_3_-TPD) was applied to evaluate the acidity. There are two types of the acidic classification as weak acidic sites with desorption peaks under temperature of ca. 200 °C, and medium to strong acid sites with desorption peak between 200 and 500 °C^30,31^. The NH_3_-TPD profiles for all catalysts are presented in Fig. 6.

The results indicated that NH_3_-TPD profiles of all spent catalysts were similar with the one obtained from the fresh catalyst at the weak acid site regime. However, the change in medium and strong acid sites regime was observed. As seen, the MCF-CSP300 exhibited the lowest amounts of medium and strong acid sites among other catalysts. In fact, the decrease in acidity can be proved by calculation of the amount of acid sites (µmole/g) as listed in Table 3. It was found that MCF-C SP300 exhibited the lowest acidity with the value of ca. 467.32 µmole NH_3_/g cat, whereas that from the MCF-C as fresh catalyst was 675.61 µmole NH_3_/g cat. This indicated that the presence of coke formation resulted in decreased acidity via obscuring on the coverage of active sites, which was probably changed to be inactive sites^32–34^ as illustrated in Scheme 1, it directly affected to either the lower ethanol conversion or yield of acetaldehyde. Therefore, the avoiding from the coke formation should be operated at high temperature of 400 °C for MCF-C catalysts on ethanol dehydrogenation. Additionally, basicity and its strength were also investigated the effect of catalyst deactivation on the active sites as basic sites.

**Table 3:** Acidity and acid strength of the fresh and spent catalysts obtained from NH_3_-TPD.

Catalysts	Amount of acid site (µmole NH_3_/g cat.)*		
	Weak acid sites	Medium-strong acid sites	Total acid sites
MCF-C	43.48	632.13	675.61
MCF-C SP300	27.68	439.64	467.32
MCF-C SP350	31.22	464.95	496.17
MCF-C SP400	35.96	579.56	615.52

*Amounts of acid sites of catalyst were calculated by NH_3_-TPD (employ of Fityk program evaluation).

Consequently, carbon dioxide temperature-programmed desorption (CO_2_-TPD) was utilized to determine the basicity of spent catalysts.

**Figure 7:**
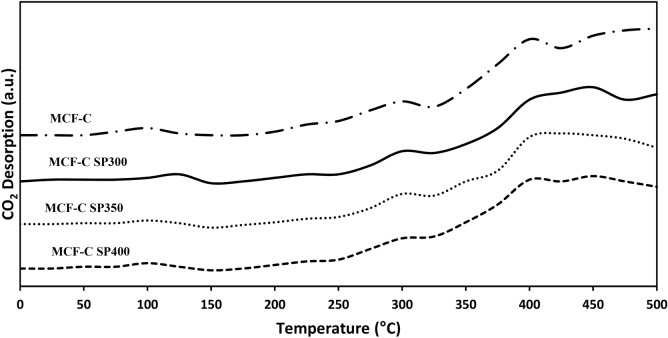
TPD-CO2 profile of the fresh and spent catalysts.

Two species of basicity were differentiated from desorption peak with below temperature of ca. 200 °C for weak basic sites and 200–500 °C for medium to strong basic sites^11^. The characteristics of CO_2_-TPD profile of each spent catalysts are exhibits in Fig. 7. This evidence suggested that all weak basic sites regime of spent catalysts slightly changed from the fresh catalyst, which was indicated that the occurrence of the coke had delicately effect to weak basic sites. Meanwhile, the medium to strong basic sites regime of spent catalysts also softly changed from fresh catalysts, especially MCF-C SP400. It could be indicated that the high temperature as 400 °C might provide appropriate condition for ethanol dehydrogenation with long period operation due to well maintain the basicity. Practically, the basicity was determined the amounts of basic sites (µmole/g) as represented in Table 4. The results indicated that the lowest loss in total basic sites was found at MCF-C SP400 with value of ca. 823.75 µmole CO_2_/g.cat from fresh catalyst as 903.52 µmole CO_2_/g.cat. This could be implied that the decrease of the basicity was involved with the appearance of the coke, which covered on the active sites as basic sites, and then it became the inactive sites, especially for MCF-C SP300 at the low temperature.

**Table 4:** Basicity and basic strength of all samples obtained from CO_2_-TPD.

Catalysts	Amount of basic site (µmole CO_2_/g cat.)*		
	Weak basic sites	Medium-strong basic sites	Total basic sites
MCF-C	61.39	842.13	903.52
MCF-C SP300	51.78	709.53	761.31
MCF-C SP350	53.02	729.11	782.13
MCF-C SP400	51.97	771.78	823.75

*Amounts of basic sites of catalyst were calculated by CO_2_-TPD (employ of Fityk program evaluation).

In order to understand the relationship between the activity and chemical properties, especially up on the basicity and acidity, the comparative results are lists on Table 5. It was found that the ethanol conversion slowly changed at high operating temperature for MCF-C SP400 owing to the low coke deposition, which was corresponding to the low percentage change in the amount of basic and acid sites. Thus, the reason of the higher catalytic activity at higher temperature was less coke deposition. In addition, at the high operating temperature, some cokes such as aliphatic coke type can be volatized^35^.

**Table 5:** The relationship between the catalytic activity and chemical properties as basicity and acidity base on operating time for 12 h.

Catalysts	Ethanol conversion change (%)	Amounts of basic sites change (%)	Amounts of acid sites change (%)
MCF-C SP300	63.17	15.74	30.83
MCF-C SP350	60.16	13.43	26.56
MCF-C SP400	22.23	8.82	8.89

**Figure 8:**
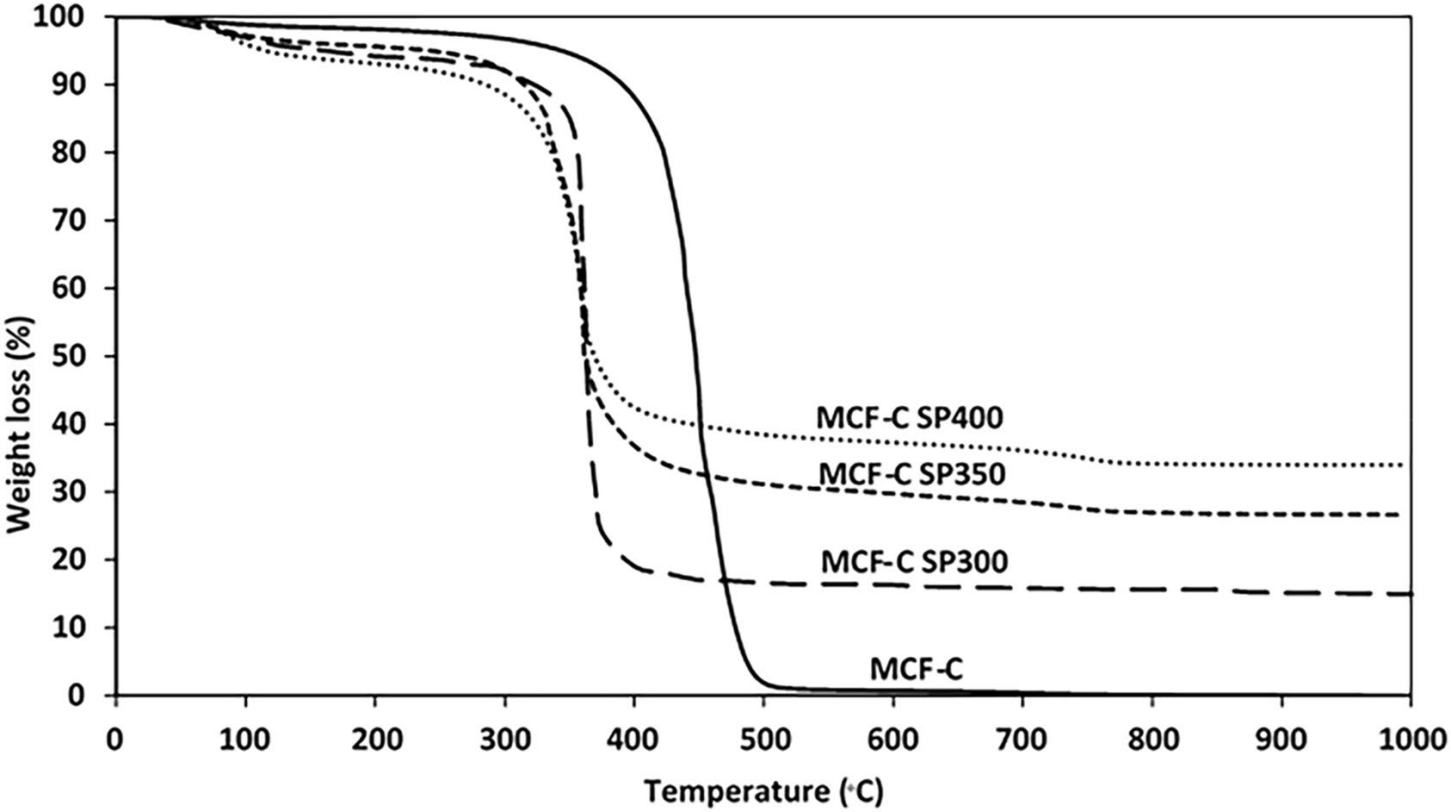
Thermogravimetric analysis (TGA) of material corresponding to each stability testing conditions of MCF-C under air atmosphere.

**Figure 9:**
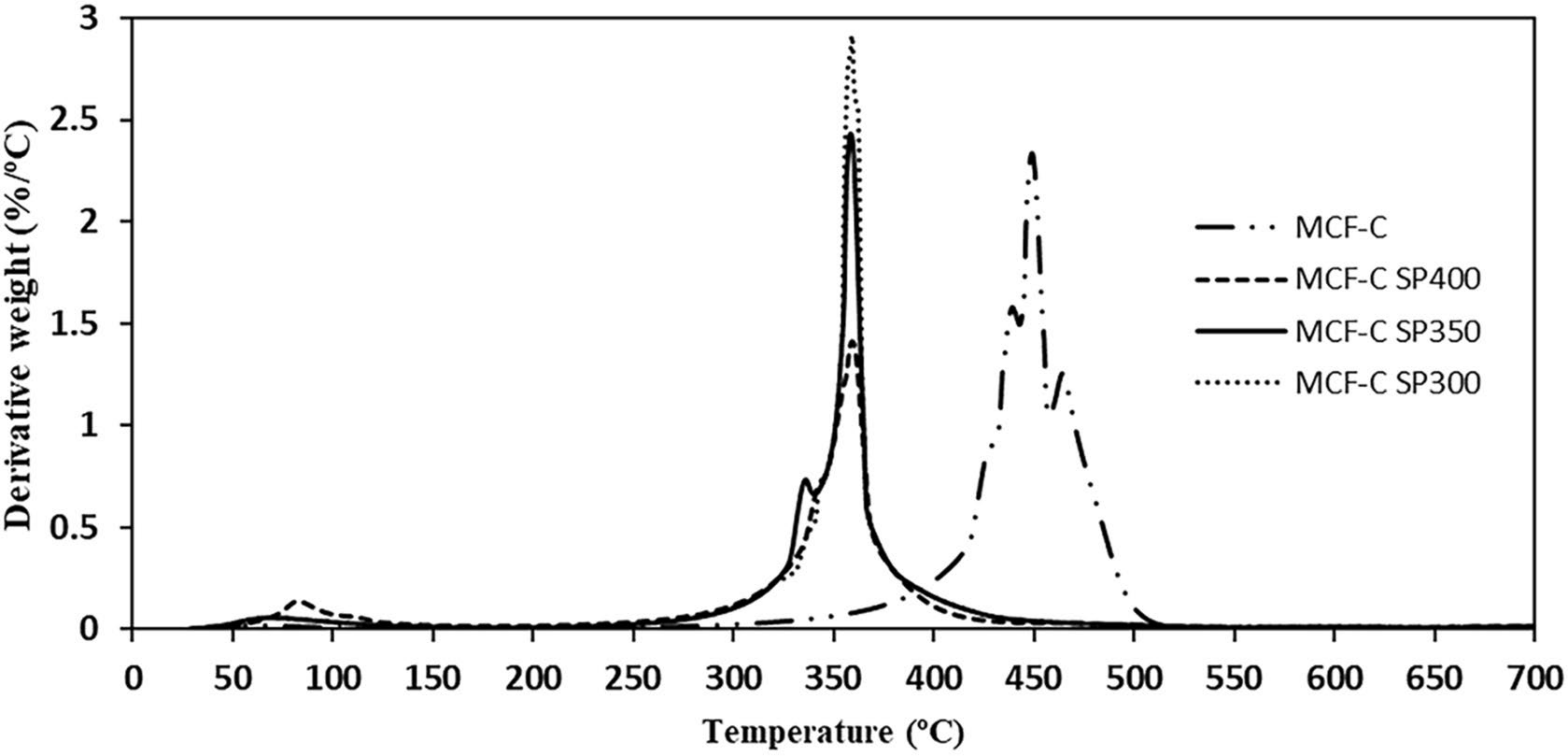
Differential thermal analysis of the fresh and spent catalysts.

The quantitative analysis of the coke formation was examined with thermal gravimetric analysis (TGA) was used to investigate the coke formation by observing the weight loss, which was operated under air atmosphere as shown in Fig. 8. TG curves of the fresh MCF-C catalyst exhibited the weight loss at 450 °C with value of 95.7%, indicating the characteristic of the mesocelluar foam carbon as reported in previous study^13^. After the reaction test for 12 h, all spent catalysts including MCF-C SP300, MCF-C SP350, and MCF-C SP had sharply declined weight loss at temperature of ca. 370 °C, which differed from the fresh MCF-C catalyst. Based on TGA, all spent catalysts mostly contained the aliphatic and aromatic coke from agglomeration of ethylene^36^, and the other parts of coke formation might be from the other by-products such as ethyl acetate with oxygen-containing functional group as supported by IR result. Furthermore, the highest percentage of weight loss of TGA curves was found on MCF-C SP300 with value of ca. 77.3% followed by MCF-C SP350 and MCF-C SP400, respectively. This indicates that MCF-C SP300 exhibited the largest amount of coke formation resulting in decreased pore volume. In addition, this result can be confirmed with differential scanning calorimetry (DSC) technique showing increased area below curve, which is related higher coke content as shown in Fig. 9. This could be point that the coke formation favored at low temperature as MCF-C SP300 for ethanol dehydrogenation.

## Conclusion

In this research, the deactivation of mesocellular form carbon (MCF-C) catalyst during ethanol dehydrogenation to acetaldehyde was investigated under different operating temperatures. The lowest catalytic activity with MCF-C was found at low temperature of 300 °C due to the highest coke formation, which directly affected deactivation of catalyst. The presence of the coke in MCF-C not only decreased the pore volume and surface area, but also decreased the acidity of catalyst. In addition, the pore blockage also retarded the mass transfer of reactant and product inside the pores. In contrary, the reaction temperature of 400 °C was softly deactivated the catalyst due to less coke formation, which preserved the catalytic activity without a significant change in ethanol conversion. Thereby, the increasing of reaction temperature in ethanol dehydrogenation to acetaldehyde using MCF-C as catalyst significantly provided either lower deactivation of catalysts or higher catalytic activity.

## Supplementary Information

Supplementary Information.

## Data Availability

The authors declare that all relevant data are within the paper.
