# Supplementary material for: Study of deactivation in mesocellular foam carbon (MCF-C) catalyst used in gas-phase dehydrogenation of ethanol
Source: Sci Rep. 2021 Jun 3;11:11683. doi: 10.1038/s41598-021-91190-7 (PMC8175389; doi:10.1038/s41598-021-91190-7)
Supplement: Supplementary file 1 — Supplementary Information. [file 41598_2021_91190_MOESM1_ESM.docx]

**Supplementary**

1. **Table S1**. The selectivity of acetaldehyde of each operating temperature with time on stream.

**Table S1**. The selectivity of acetaldehyde of each operating temperature with time on stream.

| Time (h) | Selectivity of acetaldehyde (%) | | |
| --- | --- | --- | --- |
|  | 300 °C | 350 °C | 400 °C |
| 1 | 98.32 | 91.85 | 93.88 |
| 2 | 99.85 | 90.57 | 93.54 |
| 3 | 92.55 | 91.38 | 87.19 |
| 4 | 94.11 | 94.39 | 87.50 |
| 5 | 95.72 | 88.22 | 85.52 |
| 6 | 93.62 | 86.72 | 88.17 |
| 7 | 92.15 | 88.06 | 85.74 |
| 8 | 94.47 | 94.14 | 85.09 |
| 9 | 91.65 | 91.025 | 87.22 |
| 10 | 98.69 | 98.34 | 87.46 |
| 11 | 82.19 | 95.76 | 85.38 |
| 12 | 96.77 | 96.58 | 86.82 |
